# O5-4 Technology-based physical activity interventions acceptability in obese females: a latent profile transition analysis

**DOI:** 10.1093/eurpub/ckac094.036

**Published:** 2022-08-29

**Authors:** Meggy Hayotte, Guillaume Martinent, Véronique Nègre, Pierre Thérouanne, Fabienne d'Arripe-Longueville

**Affiliations:** 1 Université Côte d'Azur, LAMHESS, France; 2 Laboratoire sur les Vulnérabilités et l'Innovation dans le Sport (L-VIS), Université Claude Bernard Lyon 1, Lyon, France; 3 Centre Spécialisé Obésité PACA Est, Pôle DARE, Centre Hospitalier Universitaire de Nice, membre de l'Université Côte d'Azur, Nice, France; 4 Université Côte d'Azur, LAPCOS, France

**Keywords:** Obesity, physical activity, acceptability, information and communication technology, latent profile transition analysis

## Abstract

**Background:**

Technology-based physical activity interventions (TbPAI) have recently been shown to be effective for care of obese women (Cotie et al., 2018). Therefore, it is necessary to assess the acceptability of TbPAI to ensure dissemination and usage in the treatment of obesity (Venkatesh et al., 2012).

As such, the purpose of this study was to: (1) identify acceptability profiles of three TbPAI in obesity care (e.g., active video games, mobile applications, videoconferencing); (2) examine the issues of consistency or change of acceptability profiles for the same individual across the three technology; and (3) explore whether technology acceptability profiles were associated with motivation for physical activity (PA), general causality orientations for PA and sociodemographic data.

**Methods:**

Three hundred and twelve women with a mean age of 30.7 *(SD*=7.1) years, and a mean BMI of 34.9 (*SD*=9.2) kg/m^2^ were recruited from health services that provide obesity management. Enrolled participants completed an online survey including the following measures: motivation for PA, general causality orientations for PA, TbPAI acceptability for the three selected technologies based on the UTAUT2 model (Venkatesh et al., 2012), and sociodemographic data. Ethical approval was gained by local committee, and informed consent were obtained from the participants before data collected. We used a Latent Profile Transition Analysis (LPTA) approach.

**Results:**

A 2-class model (high and low acceptability) best described latent classes for each technology. Acceptability profiles changed over the technologies. Among our sample, only 8.0% (n = 25) of women have low acceptability of all technologies, and 57.7% (n = 180) have high acceptability of all technologies. The covariate effect estimates showed significant effects of: (a) age and control causality orientation on video games acceptability profiles, (b) intrinsic motivation and impersonal causality orientation on videoconferencing profiles, and (c) control and impersonal causality orientations on mobile applications profiles.

**Conclusions:**

A LPTA approach may prove usefulness in understanding TbPAI acceptability in the obesity treatment and have implications for implementation and dissemination of such technologies.

